# Dystonic Cerebral Palsy Phenotype Due to GNAO1 Variant Responsive to Levodopa

**DOI:** 10.5334/tohm.746

**Published:** 2023-04-03

**Authors:** Luiz Felipe Vasconcellos, Vinicius Pinheiro Soares, Lucas Leroux de Ricchezza

**Affiliations:** 1 Institute of Neurology Deolindo Couto –Federal University of Rio de Janeiro, BR

**Keywords:** cerebral palsy, dystonia, genetic, GNAO

## Abstract

**Background:**

Cerebral palsy (CP) should not be considered a *diagnosis*, but rather a syndrome related to several etiologies, including, but not limited to, neurological sequelae of a perinatal brain injury.

**Case report:**

24-years-old man with dystonia and delayed motor and cognitive development had been previously diagnosed with CP. Molecular genetic testing identified a heterozygosity variant in GNAO 1 gene. A therapeutic trial with levodopa was started, with improvement of dystonia.

**Discussion:**

GNAO1 gene variant disorders share similarities with other causes of CP syndrome, and thus investigation of this variant should be included in instances of CP syndrome without a clear history of previous perinatal brain injury. GNAO1 dystonic phenotype (DYT-GNAO1) should be considered as dopa-responsive dystonia in some cases.

## Introduction

The meaning of cerebral palsy (CP) has changed over time. Currently, CP should not be considered a diagnosis, but rather a syndrome related to several etiologies, including, but not limited to, neurological sequelae of a perinatal brain injury [1]. Some genetic disorders may also present as a CP syndrome. Since guanine nucleotide-binding protein 1 (GNAO1) gene variant related disorders share many similarities with other causes of CP syndrome, investigation of this variant should be considered when there is a suspicion of genetic form of CP syndromes [2]. We presented a case previously diagnosed with CP of unspecified cause, in which genetic analysis revealed a pathogenic variant of GNAO1 gene.

## Case report

**Figure 1:**
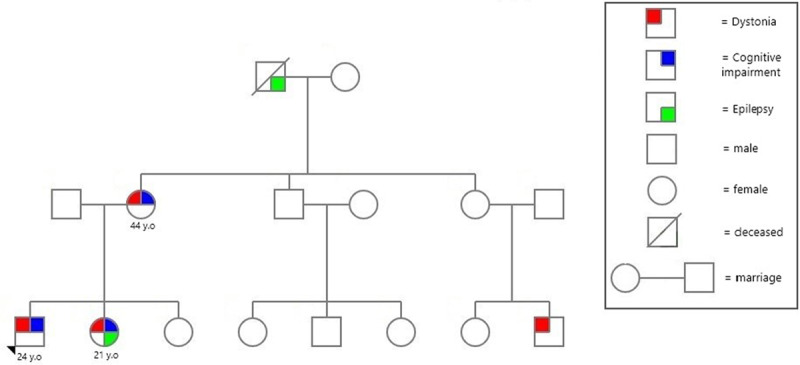
Family Pedigree.

A 24-years-old man presented with progressive gait disorder and generalized involuntary movements beginning at approximately 14-years-old. He had previously received a diagnosis of cerebral palsy (CP) of unspecified cause with delayed motor and cognitive development. Family history, as shown in Figure 1, revealed that his maternal grandfather, now deceased, had epilepsy without dystonia nor cognitive impairment, his mother has generalized dystonia and cognitive impairment, and his sister, also previously diagnosed with CP, has both epilepsy and generalized dystonia, as well as cognitive impairment.

**Figure 2:**
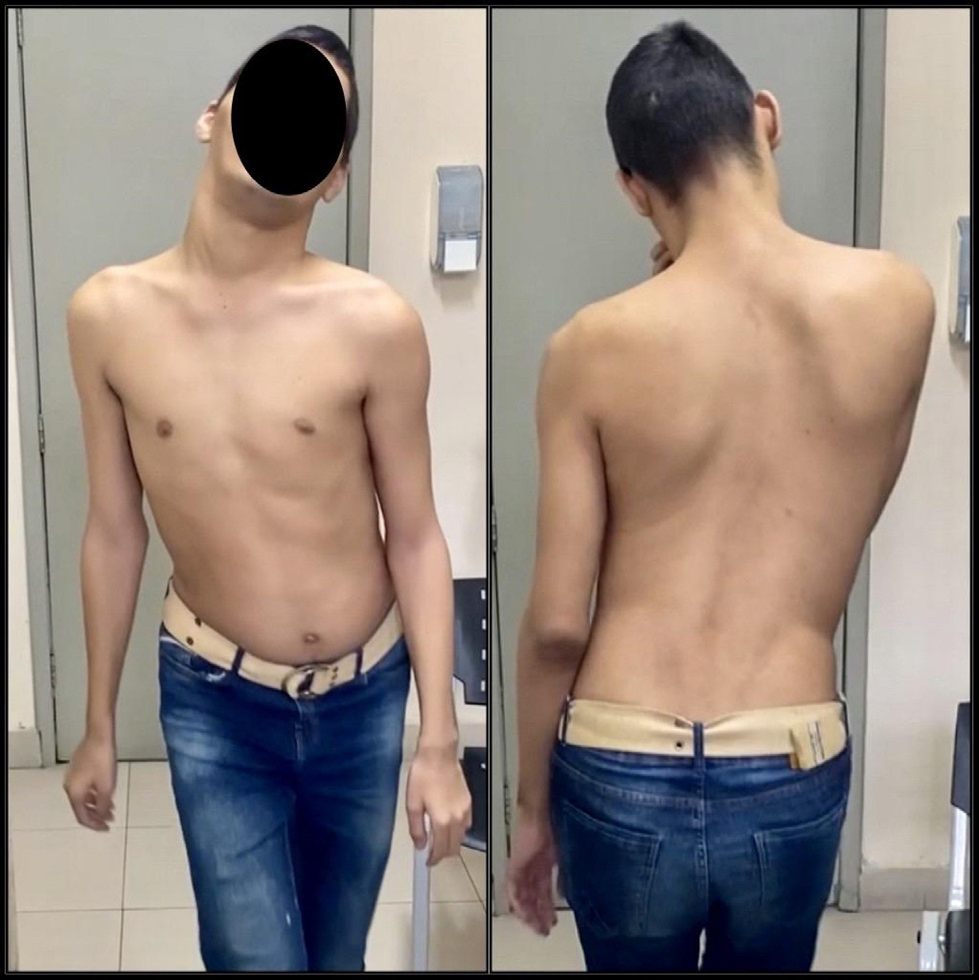
Cervical and truncal dystonia related to GNAO 1 variant.

Neurological examination showed generalized dystonia with a cervical-truncal predominance pattern (Figure 2 and video-part 1). The patient could not communicate verbally, but was capable of understanding and obeying simple commands. No dysmorphic features was observed.

**Video Part 1:** Generalized dystonia, with truncal predominance before levodopa trial. Part 2: Improvement of dystonia was observed after 300 mg/day of levodopa.

Brain MRI and electroencephalogram were unremarkable.

Molecular genetic testing identified a variant, in heterozygosity, in the splicing acceptor site of GNAO1 gene(c.724-8G>A). The mother and sister were subsequently tested, and the same variant was identified.

A therapeutic trial with levodopa was started with mild but noticeable improvement of dystonia (Video-part 2).

## Discussion

Several genes have been associated with dystonic-CP mimics: NKX2-1, ADCY5, FOXG1, GNAO1 among others [1]. Considering them in the differential diagnosis of cryptogenic or familial CP syndrome may impact prognosis, genetic counseling, and treatment of these patients.

GNAO1 related disorder was first reported in epileptic encephalopathy (Ohtahara syndrome). More recently, however, there have been reports of movement disorders (MD), epilepsy and epilepsy-MD overlap phenotypes [2]. This clinical heterogeneity is attributed to biochemical aspects of cAMP: variants resulting in gain of function are associated with MD, while loss of function is associated with epilepsy [5].

Among GNAO1 related MD phenomenology, patients most commonly present dystonia, followed by dyskinesia and chorea [6]. Our patient present dystonia, the most common MD related to GNAO1.

GNAO1 related disorders are inherited in an autosomal dominant pattern, although reports of several affected family members as in the present case, are rare; most of the published literature describe *de novo* mutation [2,3]. Missense variant is the most common related GNAO1 genotype, while splicing site variant as documented in this patient, were found in only two previously case reports [4].

Medical management of GNAO1 related dystonia (DYT-GNAO1) includes anticholinergics, levodopa, tetrabenazine, clonazepam and gabapentin, and response to medical treatment is variable. Surgery (i.e. Deep Brain Stimulation[DBS]) has also been attempted, with improvement in most cases [3,4], including as and intervention to a life-threatening MD emergency [7].

Yang et al. reported two cases with the same variant as the current report (c.724-8G>A) in a Chinese cohort, also with dystonic features, but without significant response to levodopa [4]. On the other hand, Malaquias et al. documented improvement with levodopa in a case of GNAO1 gene variant-related dystonia-chorea [8].

The current case report highlights that GNAO1 variants should be considered in the investigation of CP etiology due to its epidemiological and treatment implications, with the possibility of employing DBS or levodopa as therapeutic options. Although symptom onset typically occurs in early childhood, this report reinforces that GNAO1 variants should also be considered in the differential diagnosis of late childhood/adolescence onset dystonic encephalopathy.
